# Water-Limiting Conditions Alter the Structure and Biofilm-Forming Ability of Bacterial Multispecies Communities in the Alfalfa Rhizosphere

**DOI:** 10.1371/journal.pone.0079614

**Published:** 2013-11-04

**Authors:** Pablo Bogino, Ayelén Abod, Fiorela Nievas, Walter Giordano

**Affiliations:** Departamento de Biología Molecular, Facultad de Ciencias Exactas, Físico-Químicas y Naturales, Universidad Nacional de Río Cuarto, Río Cuarto, Córdoba, Argentina; Tel Aviv University, Israel

## Abstract

Biofilms are microbial communities that adhere to biotic or abiotic surfaces and are enclosed in a protective matrix of extracellular compounds. An important advantage of the biofilm lifestyle for soil bacteria (rhizobacteria) is protection against water deprivation (desiccation or osmotic effect). The rhizosphere is a crucial microhabitat for ecological, interactive, and agricultural production processes. The composition and functions of bacterial biofilms in soil microniches are poorly understood. We studied multibacterial communities established as biofilm-like structures in the rhizosphere of *Medicago sativa* (alfalfa) exposed to 3 experimental conditions of water limitation. The whole biofilm-forming ability (WBFA) for rhizospheric communities exposed to desiccation was higher than that of communities exposed to saline or nonstressful conditions. A culture-dependent ribotyping analysis indicated that communities exposed to desiccation or saline conditions were more diverse than those under the nonstressful condition. *16S rRNA* gene sequencing of selected strains showed that the rhizospheric communities consisted primarily of members of the Actinobacteria and α- and γ-Proteobacteria, regardless of the water-limiting condition. Our findings contribute to improved understanding of the effects of environmental stress factors on plant-bacteria interaction processes and have potential application to agricultural management practices.

## Introduction

 Bacteria living in terrestrial environments are normally organized as multicellular aggregates that develop on a variety of surfaces. These aggregates are highly complex communities, and this lifestyle (termed "biofilm") facilitates survival and resource optimization in hostile environments [1]. Potera (1996) [2] estimated that bacteria attached to surfaces and organized in biofilms are responsible for >99% of all bacterial activity in natural ecosystems. Soil bacteria occupy various microenvironments, including the rhizosphere (rich in nutrients derived from root exudates) and bulk soil (deficient in nitrogen, phosphorus, water, and other nutrients). Most soil bacteria are presumed to live as biofilms adhered to various soil surfaces (including soil particles, organic matter detritus, and roots) and to derive an advantage from this lifestyle. Protection from desiccation in water-deficient environments is considered to be a crucial advantage for rhizobacteria [3,4]. Most naturally occurring biofilms are taxonomically and functionally complex assemblies consisting of multiple bacterial species [5]. Little is known regarding the composition and functioning of biofilms in the soil [6] because of difficulties in studying the lifestyles of bacteria in edaphic microenvironments [7].

 The rhizosphere is the soil niche influenced by plant roots [8]. It is a dynamic and complex microenvironment characterized by a wide variety of interactions between bacteria and plants. Rhizosphere colonization depends on migration of bacteria from the bulk soil to rhizospheric soil that is tightly associated with plant roots. Bacteria must have the ability to establish themselves as microcolonies in order to be successful in this microenvironment [9]. Because of the essential role of biofilm development in bacterial survival and physiology, these bacterial communities must establish themselves as a multispecies biofilm at the rhizospheric level [10-12]. Biofilms are the primary structures from which bacteria play their roles in nutrient cycling [8], interactions (either beneficial or deleterious) with plants and other eukaryotes [13], reduction of biotic or abiotic plant stress factors [14], and enhancement of agricultural productivity [15]. Because they depend on organic materials derived from plant roots, rhizospheric bacterial communities are abundant, diverse, and subject to variability as a function of fluctuations in environmental factors such as water availability [16].

 Terrestrial bacterial communities are exposed to various environmental stressors, of which limited water availability is typically the most critical and has the greatest effect on survival and activity of these communities [17]. The availability of water in soils (water potential, ψ) depends on dissolved solutes (osmotic potential) and characteristics of the matrix environment (matric potential; water retention force on the ground) [18]. These two potentials represent different types of water deprivation that may affect bacterial physiology in different ways. Our understanding of the mechanisms used by bacteria to grow and survive in environments subject to desiccation remains limited and fragmentary.

 Degradation of soil quality resulting from desiccation and salinity is one of the most severe and widespread problems in modern agriculture and has been estimated to affect ~40% of potentially cultivable land worldwide [19]. The impact of these environmental stressors on soil bacteria is often dramatic [20,21]. For example, desiccation and salinity inhibit legume-rhizobia interactions and associated biological nitrogen fixation. Biofilms of *Pseudomonas putida* were shown to undergo changes in architecture and exopolysaccharide (EPS) composition to create a more hydrated microenvironment in response to water-limiting conditions [6].

 Many studies have addressed the effects of desiccation on the survival, storage, and application of inoculants [22-24], but very few have focused on soil bacteria. The impact of drying on rhizobacteria is therefore poorly understood, and many questions regarding the physiological responses of soil bacteria (including biofilm formation ability) remain unanswered. The tolerance of rhizobia to desiccation may be an indirect result of cellular adaptation to osmotic, thermal, and oxidative stresses [25]. Overlap may exist among the mechanisms of tolerance to these stresses, resulting in redundancies of the regulatory pathways responsible for general and specific responses to stress [26]. There is no evidence to date of biochemical or physiological impacts on biofilm formation by soil bacteria exposed to water-limiting conditions.

 Legumes are ecologically and economically important because of their ability to fix atmospheric nitrogen through their symbiotic relationship with rhizobia; this ability can reduce fertilizer use and environmental pollution [27]. *Medicago sativa* L. (alfalfa) is an important legume species widely cultivated in temperate areas as high-quality forage for livestock. It is also a commonly used model for studies of symbiosis with the nitrogen-fixing bacterium *Ensifer meliloti* [28,29] and a key agricultural crop in crop rotation and land restoration practices [30]. A number of genetic studies have been conducted using *E. meliloti* strains obtained from root nodules [31-34], but few have utilized bacteria recovered from alfalfa rhizospheric soil [35,36]. The characterization of bacterial communities associated as biofilm "microconsortia" in the alfalfa rhizosphere is highly desirable to help clarify the role of changes in such communities under water-limiting conditions.

 The majority of studies on bacterial community structure have focused on effects related to plant traits, agricultural management, or soil properties [37-39]. Few studies have assessed the effects of water-limiting conditions on bacterial communities established as biofilms in the alfalfa rhizosphere, and none have addressed the whole biofilm-forming ability (WBFA) or diversity of such microconsortia. Bacteria-plant interactions are crucial for a variety of ecosystem processes. It is therefore important to elucidate the structure and function (e.g., whole biofilm development) of bacterial rhizospheric communities exposed to water-limiting conditions in order to better understand the mechanisms involved in crop plant growth, adaptability to stressful conditions, and responses to agricultural practices.

 We studied the size, WBFA, structure, and species composition of cultivable bacterial communities established as biofilm structures (microconsortia) in the alfalfa (*M. sativa*) rhizosphere under three water availability conditions: nonstressful (control), desiccation, and saline. These communities were analyzed at the physiological level by measuring their biofilm-forming ability (BFA) and at the structural level by using *16S rRNA* gene analysis (ribotyping and sequencing). This study is one of the first to evaluate the environmental and physiological factors that interact with and affect alfalfa-associated rhizobacteria.

## Materials and Methods

### Soil and sampling procedure

 The soil sampled was a typical haplustol, loam/sandy loam, well drained and prone to desiccation. Samples of the top portion of bulk soil (0-20 cm) were collected during the winter season (June 2012) from various locations in a forage field that is typically used for cultivating alfalfa (for feeding livestock) with rotation to grain crops such as maize, wheat, and oats. The field is in the Bulnes locality in the agricultural region of the Argentinean Pampas, in the dairy farming area in southern Córdoba province (33°31’41’’S, 64°39’00’’W). The study was conducted on a privately owned field with the permission of the land owner, and did not involve endangered or protected species. Each soil sample was immediately mixed, sieved to remove plant detritus, placed in a sterile plastic bag, transported to the laboratory in an ice cooler, and stored at 4 °C until analysis. The humidity content (H%) at the time of sampling was 6.7%. The electrical conductivity (EC) was 0.17 dS m^-1^.

### Soil treatments

 Experimental pots were filled with 2 kg of soil each. Alfalfa seeds (Pampeana Cordoba cultivar) were sown so as to uniformly cover the pot surface and to reflect the typical field density of 25 kg alfalfa seed per hectare. Potted plants were grown in a greenhouse under controlled conditions of 16/8 h light/dark at 28/24 °C. The three experimental treatments, with 4 pots per treatment, were: (1) nonstressful conditions (control) (regular watering; final H 13.4%; EC 0.17 dS m^-1^) (2), desiccation stress (limited watering; final H 4.2%; EC 0.18 dS m^-1^), and (3) saline stress (regular watering; final H 12.2%; added with 2.5 g NaCl per kg soil; EC 2,54 dS m^-1^; resulting in a slightly saline soil). The rhizospheric soils obtained from these 3 treatments were termed respectively as CRS (Control Rhizospheric Soil), DRS (Desiccated Rhizospheric Soil), and SRS (Saline Rhizospheric Soil).

### Sampling of rhizospheric soils

 Fifty days after germination (F2 growth stage; preflowering) all plants growing in a given pot were removed to obtain rhizospheric soil samples for each condition (CRS, DRS, SRS). Suspensions of rhizospheric soils were obtained as described previously [40], with some modifications. In brief, roots were (i) shaken manually to carefully separate soil not tightly adhered to the root systems, (ii) placed in a sterile Erlenmeyer flask containing 100 ml phosphate-buffered saline (PBS; pH 7.4), and (iii) subjected to rotary shaking at 150 rpm for 1 h. Bacterial counting and isolation were performed on "rhizospheric soil suspensions" following removal of roots. Our rhizospheric soil suspensions contained bacteria from soil particles that were tightly associated with roots as well as bacteria adhered to root surfaces. We assume that the bacteria in the suspensions were representative of the bacteria present in the multispecies biofilm community. Following the microbiological analyses, the suspensions were dried and weighed so that results could be expressed per g rhizospheric soil.

### Bacterial counting and isolation

 Immediately after the rhizospheric soil suspensions as above were obtained, a series of 10-fold dilutions were made and placed on dishes with nonselective Nutrient Agar (NA) medium supplemented with 60 µg ml^-1^ cycloheximide to inhibit fungal growth. Three replicate plates were made for each dilution. The plates were incubated at 28 °C for 2-7 days, and bacteria were counted. The number of bacteria was expressed as log_10_ colony-forming units (CFU) per g dried rhizospheric soil. For each treatment, a total of 95 colonies were isolated randomly from the plate counts of the highest dilution, representing the most abundant bacterial members of each rhizospheric community. Selected strains were grown on LB medium [41] until the late exponential phase and stored at -80 °C in 20% glycerol solution.

### Biofilm-forming ability (BFA) assay

 BFA was determined macroscopically by a quantitative assay using 96-well microtiter dishes as described by O'Toole and Kolter (1998) [42]. Bacterial preinocula were grown in 2 ml TY medium [43] and incubated with agitation for 48 h at 28 °C. The cultures were diluted with fresh medium to give an OD_620_ of 0.01, and 100 µl of each bacterial suspension was added to each well and incubated for 24 h at 30 °C. Bacterial growth was quantified by measuring the absorbance of planktonic cells in each well at OD_620_ with a MicroELISA Auto Reader (Series 700 Microplate Reader; Cambridge Technology). Planktonic cells were then removed, each well was washed 3 times with saline solution, and cells adhered to the polystyrene support (i.e., biofilm) were stained with 180 µl crystal violet (0.1%) for 15 min. The wells were rinsed repeatedly with distilled water, and biofilm formation was assayed by addition of 150 µl of 95% ethanol. The OD_570_ of solubilized crystal violet was measured with a MicroELISA Auto Reader as above. Parallel, sterile control cultures were established in TY medium. Relative BFA was calculated as OD_570_/OD_620_ (biofilm quantified by staining with crystal violet relative to planktonic growth measurement). The above methodology was employed for various purposes. For the 3 types of bacterial communities (CRS, DRS, SRS), each consisting of ~95 strains, we obtained a WBFA value based on the mean value of 96-well microtiter dishes obtained from five replicates. We ordered the strains according to their BFA values and selected subpopulations of 15 strains with high BFA (HBFA) and 15 strains with low BFA (LBFA) from each community for genotypic characterization, as described below.

### Characterization of alfalfa rhizospheric communities by 16S rRNA gene analysis

 A total of 90 bacterial strains (30 for each community or treatment, divided into subpopulations of 15 HBFA and 15 LBFA strains as described above) were characterized by Amplified Ribosomal DNA Restriction Analysis (ARDRA). Bacterial genomic DNA was extracted from each isolate using a Genomic DNA Purification kit (Thermo Scientific/ Fermentas Life Science, USA), according to the manufacturer’s instructions. Primers fD1 (5´-AGAGTTTGATCCTGGCTCAG-3´) and rD1 (5´- AAGGAGGTGATCCAGCC-3´) [44] were used for *16S rRNA* gene amplification. Aliquots of PCR products, each ~1500 bp, were digested by restriction endonuclease HaeIII (Thermo Scientific/ Fermentas) [45]. DNA digestion fragments were separated electrophoretically on a 3% (w/v) agarose gel, stained with ethidium bromide, visualized under UV illumination, and photographed. The identification of each ribotype was associated with a particular digestion fingerprint. Diagrams of community structure were constructed based on the ribotypes found. Strains belonging to either majority unique ribotypes from a particular treatment or common ribotypes present for all 3 treatments were selected for further characterization by identification through *16S rRNA* gene nucleotide sequence analysis and study of traits related to BFA.

### 16S rRNA gene nucleotide sequence analysis

 The nucleotide sequence of *16S rRNA* gene was analyzed for a total of 13 bacterial strains from the 3 types of rhizospheric soil (strains C3, C7, C12, C29, and C35 strains from CRS; M1, M10, M29, and M32 from DRS; S2, S13, S36, and S37 from SRS) (Table 1). Direct PCR was performed with 1 µl DNA template in a 20 µl PCR reaction mixture containing the universal primers 27F (5´-AGAGTTTGATCCTGGCTCAG-3´) and 1492R (5´-TACGGTTACCTTGTTACGACTT-3´) [46]. Purified PCR products (each ~1400 bp) were sequenced with an automated DNA sequencing system (model 3730XL, Applied BioSystems, USA) by Macrogen Inc. Laboratories (Seoul, South Korea). The *16S rRNA* gene sequences were analyzed using the BLAST search program (National Center for Biotechnology Information [NCBI]; http://blast.ncbi.nlm.nih.gov/Blast.cgi) [47] to find identities among sequences. 

**Table 1:** Identities and BFA-related traits of bacterial strains isolated from various rhizospheric soils.

	***16SrRNA* gene**				**BFA-related traits**				
**Strain (Source)**	**Rt**	**GenBank accession no.**	**Most closely related sequence (accession number) (Id %)**	**Phylogenetic affiliation**	**BFA**	**Agg (%) (type)**	**Motility (cm)**	**EPS**	**Long AHL**
**C3 (CRS)**	1**^u^**	KF261554	*Agrobacterium* sp. AHL7 (AY379979.1) (99)	α-Proteobacteria	2.30 ± 0.28	29.6 ± 5.6 SA	2.55 ± 0.49^c^	-	**+**
**C7 (CRS)**	4**^˄^**	KF261555	*Microbacterium* sp. S18 (EU747700.1) (99)	Actinobacteria	1.94 ± 0.30	35.4 ± 3.4 MA	0.77 ± 0.21^a^	-	+
**C12 (CRS)**	3*	KF261556	***Rhizobium* sp. R-24658 (AM084043.1) (99)**	α-Proteobacteria	1.50 ± 0.29	37.2 ± 6.1 MA	1.83 ± 0.22^b^	-	+
**C29 (CRS)**	7^#^	KF261557	*Arthrobacter* sp. DNS10 (HQ914648.1) (99)	Actinobacteria	0.11 ± 0.03	23.8 ± 5.8 SA	-	+	**-**
**C35 (CRS)**	7^#^	KF261558	*Promicromonospora* *sp.* FFN01 (JN896618.1) (99)	Actinobacteria	0.06 ± 0.02	67.2 ± 2.0 HA	-	-	**+**
**M1 (DRS)**	3*	KF261559	*Stenotrophomonas* sp. CK6 (AJ870967.1) (100)	γ-Proteobacteria	3.40 ± 0.38	41.4 ± 5.8 A	2.88 ± 0.50^cd^	+	**-**
**M10 (DRS)**	4**^˄^**	KF261560	*M. hydrocarbonoxydans* HNR08 (EU373354.1) (99)	Actinobacteria	2.19 ± 0.37	40.5 ± 7.8 A	0.79 ± 0.19^a^	-	**+**
**M29 (DRS)**	7**^#^**	KF430812	*Arthrobacter* sp. DNS10 (HQ914648.1) (99)	Actinobacteria	0.09 ± 0.02	45.3 ± 7.7 A	0.87 ± 0.23^a^	-	**+**
**M32 (DRS)**	8**^#^**	KF261561	*M. testaceum* SD9-677 (JQ660317.1) (99)	Actinobacteria	0.06 ± 0.01	24.8 ± 3.5 SA	-	-	**-**
**S2 (SRS)**	19**^u^**	KF261562	*Pseudomonas* sp. AF32 (EU680973.1) (99)	γ-Proteobacteria	2.34 ± 0.54	37.2 ± 6.0 MA	3.38 ± 0.43^d^	-	**+**
**S13 (SRS)**	3*	KF261564	*Rhizobium* sp. R-24658 (AM084043.1) (99)	α-Proteobacteria	1.02 ± 0.20	43.2 ± 5.7 A	2.95 ± 0.13^cd^	-	**+**
**S36 (SRS)**	7**^#^**	KF261565	*Arthrobacter pascens* H45 (KC934828.1) (99)	Actinobacteria	0.05 ± 0.02	49.6 ± 8.2 A	-	+	**+**
**S37 (SRS)**	25**^u^**	KF261566	*Shinella granuli* Ch06 (AY995149.1 ) (98)	α-Proteobacteria	0.05 ± 0.03	9.5 ± 1.7 BA	0.95 ± 0.13^a^	+	**-**

Rt: Ribotype. Superscript symbols in ribotype column: u: majority unique ribotype; ˄: shared ribotype for CRS and DRS strains; * shared ribotype for HBFA strains; # shared ribotype for LBFA strains. Id: Identity. Agg: Autoaggregation expressed in %. Aggregation categories based on statistical analysis: BA, barely aggregative; SA, slightly aggregative; MA, moderately aggregative; A, aggregative; HA, highly aggregative. Motility is expressed in terms of the halo diameter (cm) (see Materials and Methods). Differing letters indicate significant differences. EPS: exopolysaccharide. AHL: acyl homoserine lactone.

### Nucleotide sequence accession numbers

 The nucleotide sequences of the *16S rRNA* genes of the alfalfa rhizosphere strains C3, C7, C12, C29, C35, M1, M10, M29, M32, S2, S13, S36, and S37 determined in this study have been deposited in the GenBank nucleotide sequence database (NCBI; www.ncbi.nlm.nih.gov/genbank) under accession numbers KF261554, KF261555, KF261556, KF261557, KF261558, KF261559, KF261560, KF430812, KF261561, KF261562, KF261564, KF261565, and KF261566, respectively. The strains and their GenBank accession numbers are listed in Table 1.

### Clustering analysis

 The genetic distances among the 3 rhizospheric soils (CRS, DRS, SRS) and their subpopulations (HBFA and LBFA) were related to the ribotypes found for each condition, and dendrograms were constructed accordingly. We constructed a 2-dimensional binary matrix using a binary scoring system: 1 for presence or 0 for absence of a given ribotype for a particular condition. The distances among conditions were evaluated using the Jaccard index. Dendrograms were constructed with Infogen software, using the UPGMA algorithm [48].

### Study of traits related to BFA

 Various physiological processes related to BFA were evaluated for selected strains from each of the 3 alfalfa rhizospheric soils. Autoaggregation assays were performed as described by Sorroche et al. (2010) [49]. Each strain was grown for 24 h at 28 °C in TY medium. Five ml of bacterial suspension was transferred to a glass tube (10 × 70 mm) and left to settle for 24 h at 4 °C. A 0.2-ml aliquot of the upper portion of the suspension was transferred to a microtiter plate, and OD_620_ was measured (OD_final_). A control tube was vortexed at ~1500 rpm until a homogeneous bacterial suspension was obtained, and OD_620_ was measured (OD_initial_). The autoaggregation percentage was calculated as 100[1 – (OD_final_/OD_initial_)].

 Motility (swimming assay) of each strain was evaluated by inoculation (center puncture) of a plate containing reduced 1/10 TY medium with 0.3% agar [50] through visualization of homogeneous halos of bacterial motility. The plates were incubated for 3 days at 28 °C and halo diameters (indicators of motility) were measured in cm.

 EPS production was assessed qualitatively based on fluorescence under UV exposure of EPS-producing strains grown on LB medium containing 0.02% calcofluor white [51].

 The production of quorum-sensing molecules was studied qualitatively using the biosensor *Chromobacterium violaceum* CV026 for detection of acyl homoserine lactones (AHLs) with short acyl chains [52] and the biosensor *Agrobacterium tumefaciens* NTL4 (pZLR4) for detection of AHLs with long acyl chains [53].

### Statistical analysis

 The values presented are means of replicate experiments; the number of replicates varied depending on the experiment. The data were subjected to one-way analysis of variance (ANOVA) followed by comparison of multiple treatment levels using *post hoc* Fisher’s Least Significant Difference (LSD) test. To evaluate the overall relationships of treatments, subpopulations, ribotypes, phylogenetic affiliations, and BFA-related traits, we performed a multivariate study with principal components analysis (PCA). Statistical analyses were performed using the InfoStat software program, version 2.0 (Grupo InfoStat, Universidad Nacional de Córdoba, Argentina).

## Results and Discussion

### Determination of cultivable bacteria counts in alfalfa rhizospheric soils

 The primary lifestyle of most soil bacteria is as part of a multispecies biofilm. Soil ecosystems provide various microenvironments in which bacteria can become established as biofilms. The rhizosphere is a highly suitable location for development of the biofilm lifestyle because of the presence of root-derived nutrient sources and a biotic surface with which bacteria can actively interact [12,54]. In contrast, bulk soils provide poor or even hostile microniches for bacteria that survive through adhesion to soil particles and *a posteriori* development of a biofilm [55].

**Figure 1:**
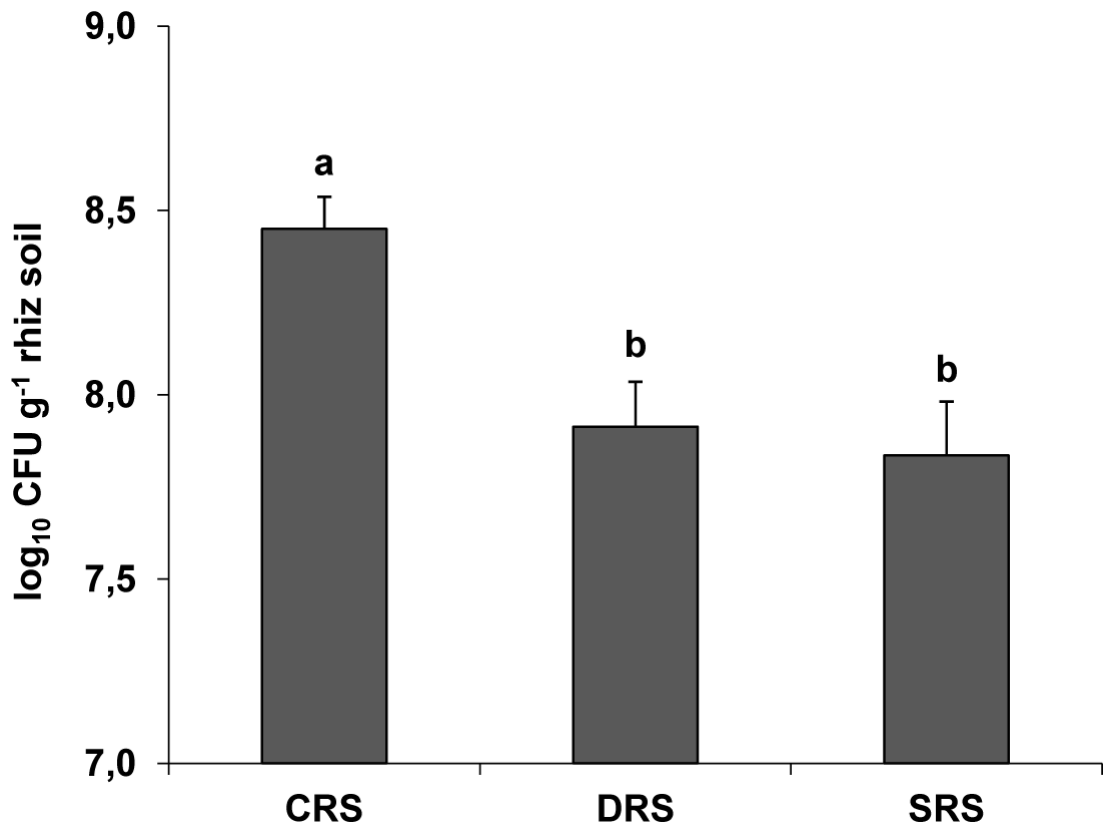
Total cultivable bacterial counts in 3 types of alfalfa rhizospheric soils. Counts of cultivable bacteria in rhizospheric soils exposed to nonstressful condition (CRS) and water-limiting conditions (DRS and SRS) are expressed as log_10_ CFU per g dry rhizospheric soil. The values and error bars are mean and S.D. of 4 replicates per treatment. Differing letters indicate significant differences (P< 0.05) between counts.

 Bacterial cell counts greater than 9.0 log_10_ CFU per g field soil have been reported in previous studies [56]. We obtained mean bacterial counts on NA medium of 7.31 ± 0.23 log_10_ CFU per g original bulk soil (data not shown), which is reasonable in view of the low organic matter content (1.03%) of the sampled field soil and the fact that we counted only the cultivable fraction of total bacteria. Our bacterial counts in bulk soil are comparable to those of cultivable bacteria obtained in previous studies on various types of soil exposed to various agricultural practices [57]. Interestingly, the bacterial counts for our 3 rhizospheric soils (CRS, DRS, SRS) (Figure 1) were significantly higher than those for the original bulk soil prior to the experimental treatments. Our findings reflect the previously described “rhizosphere effect” [58]; i.e., in comparison with bulk soil, soil environments closely associated with root systems provide a favorable microniche for microbial growth and activity because of the presence of nutrients derived from root exudates and rhizodeposits [35,59].

 Previous studies have shown that 1 g rhizospheric soil contains ~7.0-9.0 log_10_ CFU cultivable bacteria [9]. Our counts for rhizospheric soils exposed to nonstressful conditions (CRS) or water-limiting conditions (DRS and SRS) ranged from 7.7 to 8.5 log_10_ CFU per g dry rhizospheric soil (Figure 1). These values are similar to or higher than counts obtained in previous studies of rhizospheric soils from legume and nonlegume plants [57,60,61]. In particular, our counts are consistent with those obtained for cultivable bacteria in rhizospheres of legumes such as *Glycine max*, *Vigna radiata*, *Arachis hypogaea*, and *Acacia mangium* [57,62,63].

 We found that exposure to water-limiting conditions resulted in changes of bacterial counts in alfalfa rhizospheric soils. Interestingly, the number of CFU per g rhizospheric soil was lower under stress conditions (DRS, SRS) than nonstress condition (CRS) (Figure 1).

**Figure 2:**
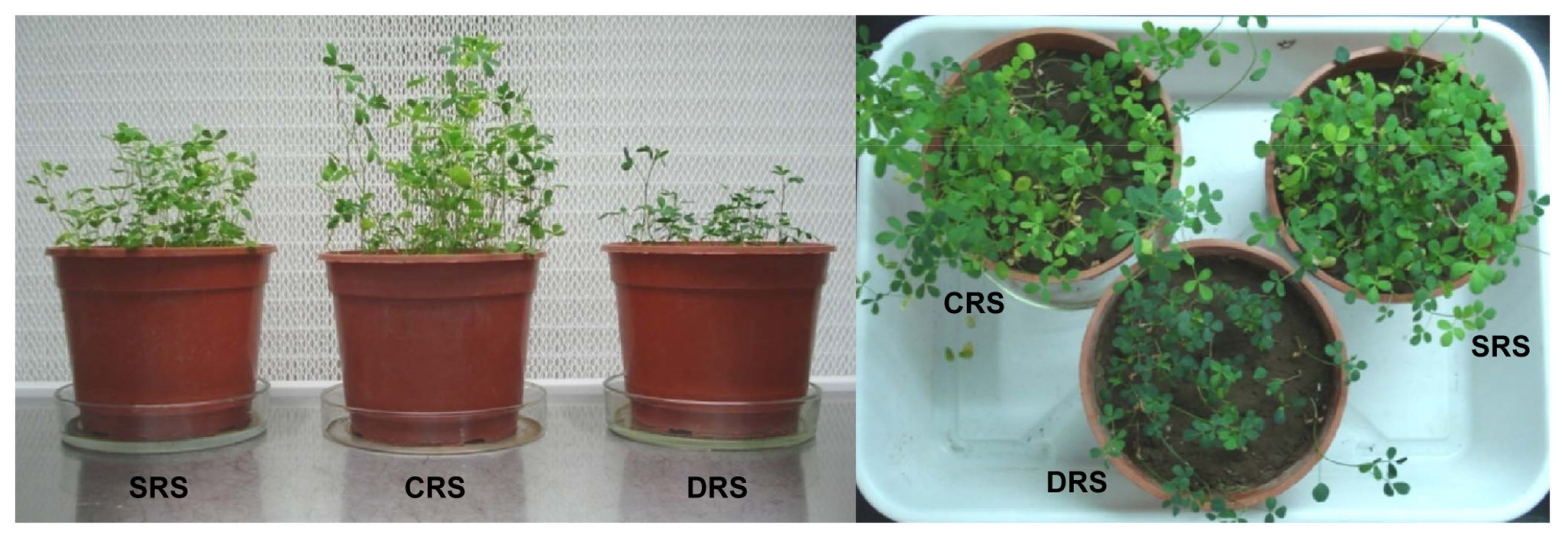
Growth of alfalfa plants exposed to 3 experimental conditions. Aerial development of alfalfa plants at the F2 stage (preflowering) under CRS, DRS, and SRS conditions.

 Exposure to water-limiting conditions clearly affected alfalfa plant development (Figure 2), although analysis of stress effects on biomass parameters was not part of our research design. Such effects were more severe for desiccation stress (DRS) than for saline stress (SRS), most likely because the degree of salinity applied was moderate (soil EC: 2.46 dS/m; slightly saline). Aerial plant development reflects root development, which was considerably lower for DRS than for SRS or CRS. The observed differences in bacterial counts may be related to the differential composition of root exudates under each condition, in view of the role of the exudates in modulating plant-bacteria interaction through alterations of bacterial metabolism, gene expression, and bacterial community structure [62,64]. Nutrient and desiccation stress have been shown to produce significant changes in the quantity and composition of root exudates [65]. Soil drying had a strong impact on total bacterial and pseudomonad counts, which were reduced by water limitation [57]; i.e., desiccation greatly reduced the number of bacterial communities. Previous studies showed higher biomass and microbial activity in environments with higher water content [66,67], in agreement with our findings.

 Rhizodeposition of alfalfa plants exposed to saline stress differs from that of nonstressed plants because increased salinity induces changes in plant hormonal balances. Studies on the effects of salinity in *Medicago truncatula* and *Lotus japonicus* indicate that a large proportion of the genome is involved in responses to high salinity and desiccation [68].

### Whole Biofilm-Forming Ability (WBFA) of 3 rhizospheric bacterial communities

 The rhizospheric niche is a dynamic microenvironment harboring a polymicrobial community affected by its own metabolic processes and by the variable composition of root exudates, which depends on the plant species and their stage of growth [69,70]. We defined rhizospheric bacterial communities as those living as multispecies biofilm microconsortia recovered from (i) bacterial suspensions obtained from soil tightly associated with alfalfa roots, and (ii) bacteria colonizing the root surface. This definition is supported by previous studies indicating that the majority of strains present in biofilms are cultivable [10].

 Many previous studies have assessed the effects of saline stress or desiccation stress on the BFA of particular bacterial strains [6,71,72]. However, effects of water-limiting conditions on BFA have never been evaluated using isolates obtained from an entire bacterial community.

**Figure 3:**
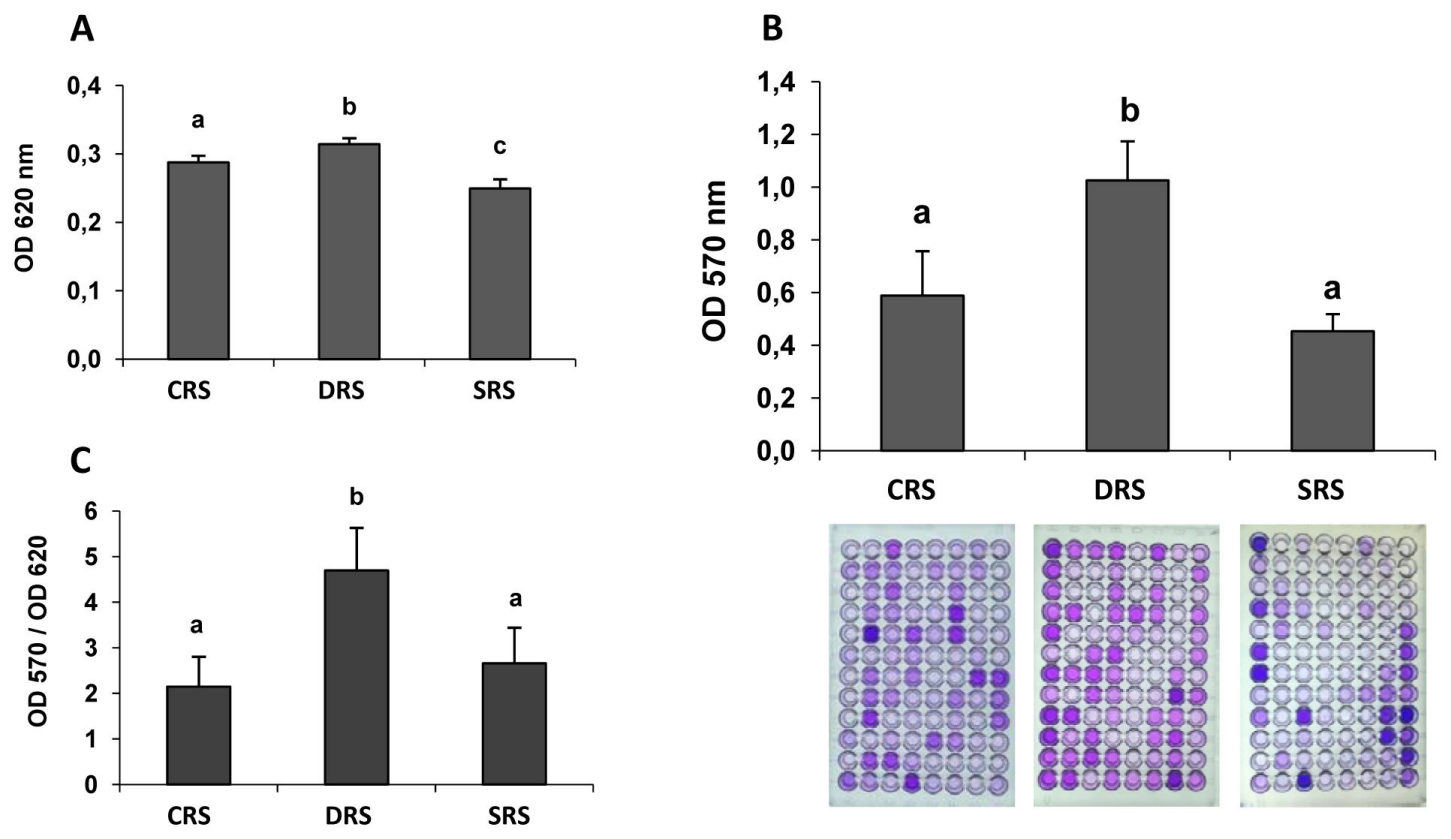
Whole planktonic growth (A), WBFA (B), and relative BFA (C) of bacterial communities from 3 types of alfalfa rhizospheric soil. The values are means of OD_620_ (planktonic growth), OD_570_ (biofilm formation quantified by staining with crystal violet), or their ratio (B/G) obtained for each plate (~95 strains) and averaged from 5 independent replicates for each treatment. The image at the bottom of Panel B shows the actual plates for each treatment. Differing letters indicate significant differences between treatments according to Fisher’s LSD test (P< 0.05).

 We obtained bacterial communities from rhizospheric soils under 3 experimental treatment (CRS, DRS, SRS), as described in Materials and Methods. Each of these communities consisted of ~95 strains isolated from the count plates of greater dilution, thus representing the most numerous members of each community. The WBFA was evaluated for each community as the mean value of 5 replicates of the BFA for each microplate, containing 95 strains per treatment. Whole growth (Figure 3A), WBFA (Figure 3B), and relative BFA (biofilm/growth ratio) (Figure 3C) were greater for the DRS community than for the CRS or SRS communities.

 The WBFA (Figure 3B) and relative BFA (Figure 3C) were almost twice as high for the DRS community as for the CRS or SRS communities. As seen in the microplate images (Figure 3B, bottom), the number of dishes having an intense violet color was twice as high for DRS as for CRS or SRS.

 These findings indicate that a decrease in soil water potential at the expense of matric potential (drying) leads to the establishment of a bacterial community in the alfalfa rhizosphere consisting of members having a greater capacity for growth under desiccation stress. This phenomenon may be related to a greater ability to exploit nutrient resources under such stress. Similarly, desiccation stress may lead to the selection of bacterial strains with greater BFA, resulting in increased survival of the strains. Previous studies have shown relationships between such an effect and the role of EPSs in the biofilm matrix; EPSs may reduce the effects of desiccation on both the survival of bacterial communities and plant growth [3,6,73,74]. Bacterial colonies isolated from air-dried soils were similar in aspect [57], consistent with our observation that desiccation stress exerts a selective effect on bacterial populations.

 It is remarkable that the SRS community showed smaller whole growth and WBFA than the CRS or DRS communities. The reduction of water potential resulting from increased soil solute content evidently operates differently from the effect of desiccation. Either osmotic or matric stress may have a different effect on the composition of root exudates and thus on the bacterial community associated with the roots. For example, the composition of flavonoid exudates from *Phaseolus vulgaris* roots was altered by saline stress [75].

 Both soil conditions and the amount and composition of rhizodeposits regulate the specificity of plant-bacteria interactions [76]. In the present study, despite the fact that the original soil and the plant under study were the same, it is possible that the root exudates produced under the 3 experimental conditions were different and the chemotactic effect on soil bacteria selected different communities that were specifically adapted to the particular condition [77]. Exposure to water-limiting conditions can affect bacterial communities indirectly through changes in plant metabolism, plant development, and the composition of rhizodeposits released to the rhizosphere. Previous studies have shown that, in response to water limitation, plants synthesize osmolytes [78] that are released into the rhizospheric soil and act synergistically with osmolytes produced by bacteria [76].

 In terms of bacterial physiology, bacteria living under water-limiting conditions (matric or solute stress) must integrate their responses to create a hydrated microenvironment to protect themselves. The association of bacterial populations as a multispecies biofilm in the rhizospheric microenvironment represents a lifestyle strategy for increasing survival under stress conditions. Desiccation stress may lead to selection of strains with higher BFA that have a survival advantage because of their ability to colonize root surfaces, whereas other mechanisms not necessarily related to BFA may play important roles in bacterial survival under saline stress conditions [19,25,76]. Osmotically stressed cells undergo structural changes of key macromolecules required for establishment and development of mature biofilms, including EPSs [79-81] and lipopolysaccharides [82-84].

### Composition and diversity of the 3 rhizospheric bacterial communities

 We studied the composition of the bacterial communities associated with each of the 3 rhizospheric soils in order to elucidate (i) the structure, diversity, and identities of the predominant bacteria in biofilms established as multispecies microconsortia in the alfalfa rhizosphere, and (ii) the effects of exposure to stress conditions on such communities. To simplify the analysis, we divided the isolates from each treatment into 2 groups of 15 strains with the highest or the lowest BFA (HBFA and LBFA subpopulations, respectively).

**Figure 4:**
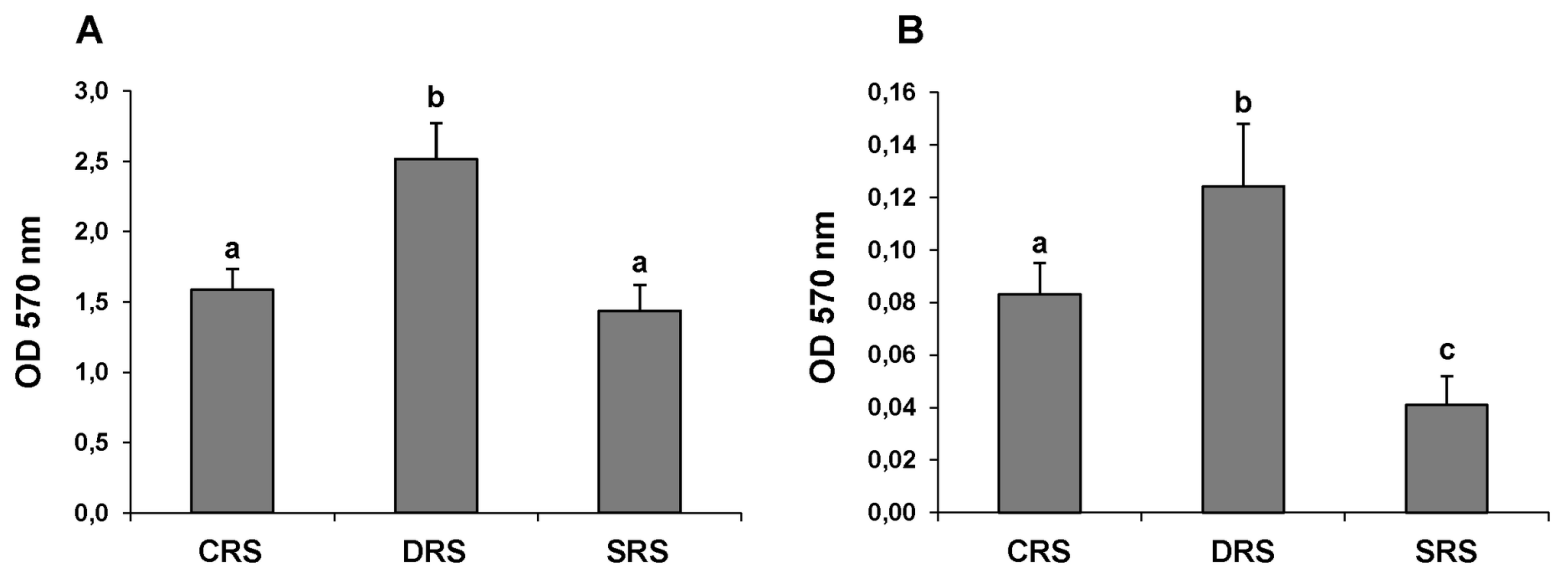
BFA of bacterial subpopulations (HBFA and LBFA) isolated from 3 types of alfalfa rhizospheric soil. BFA of 15 strains grouped as HBFA (A) and LBFA (B) subpopulations of CRS, DRS, and SRS communities as explained in the text. The bars indicate the mean value of OD_570_ (biofilm formation quantified by staining with crystal violet) from 5 independent replicates for each treatment. Differing letters indicate significant differences between treatments according to Fisher’s LSD test (P< 0.05).

 Mean BFA values for each subpopulation were compared with those of the other subpopulations and simultaneously with the WBFA value for the whole community (Figure 3). The values for the HBFA and LBFA subpopulations of the DRS community were higher than those of the CRS or SRS communities (Figure 4), indicating again that desiccation stress results in selection of strains with increased BFA.

 Regardless of the treatment, BFA values were smaller for the LBFA subpopulations (Figure 4B) and involved an important group of strains in the multispecies biofilm community. Multispecies biofilms formed in the rhizosphere of plants (in this case alfalfa) involve members of HBFA subpopulations (Figure 4A) and allow other bacterial strains without BFA to take advantage of the biofilm conglomerate and settle in a protected microenvironment. Synergistic interactions such as co-metabolism [10], cell aggregation [85], and the transfer of key determinants via conjugative plasmids [86], appear to play key roles during the development of multispecies biofilms [87,88], although most studies to date have focused on simplistic two-species associations.

 The coexistence of LBFA and HBFA strains within a community may alternatively be explained by a cooperative mechanism whereby particular bacterial strains that lack BFA in isolation gain some BFA in the presence of other strains (even low-BFA strains). This scenario could lead to the establishment of mixed biofilms that allow coexistence of strains, avoid competition [89,90], and promote synergism [91] and gene transfer [92]. In view of the complexity of edaphic microenvironments, we speculate that a combination of the two above mechanisms ("rescue" of LBFA strains by HBFA strains and cooperation among low-BFA strains) is crucial for the establishment of polymicrobial communities in plant rhizospheres.

 To study the composition of microbial communities, we used a cultivation-dependent technique based on comparison of fingerprinting by ARDRA with restriction enzyme HaeIII. The rRNA genes are essential, and their sequences are highly conserved in bacteria. The rRNA genes also have nonconserved sequences that vary among species and families [93]. ARDRA is a common tool for studying bacterial community composition and diversity [94-96]. Although it is not suitable for analysis of overall bacterial diversity from an environmental source through a single fingerprinting technique [97,98], it is useful for the study and comparison of ribotypes in large numbers of samples from differing conditions (e.g., stress conditions as in the present study). Thus, ARDRA is a good tool for ecological studies. Several previous studies have been based on the use of a single restriction enzyme with amplified rRNA genes [61,63,99,100].

 We performed a PCR-RFLP study of the most abundant strains of the HBFA and LBFA subpopulations for each treatment (CRS, DRS, SRS). We decided to conduct this laborious method (as opposed to other possible methods; e.g., *16S rRNA* gene library) because it allowed us to obtain bacterial strains for further characterization of their biofilm properties and studies of mixed biofilm mechanisms, coexistence processes (synergism, rescue, cooperation), and bacterial traits beneficial to the host plant under stress conditions.

 We selected the restriction endonuclease HaeIII because of its poor discrimination among typical rhizobacteria strains (rhizobia) [45,101], which makes it useful for distinctions at the family level, in view of the potential complexity of bacterial communities isolated from rhizospheric soils.

 The ribotypic variability of HBFA and LBFA subpopulations present in each type of rhizospheric soil was investigated following digestion of purified PCR products of *16S rRNA* genes by HaeIII. Three to 6 fragments ranging in size from 80 to 750 bp were generated among the 35 ribotypes obtained from the 90 strains evaluated, indicating a ratio of ~2.5 between the number of strains analyzed and the number of ribotypes obtained. Regardless of the experimental treatment, the bacterial strains that colonized the alfalfa rhizosphere were highly diverse, similarly to results in studies of tobacco [61].

**Figure 5:**
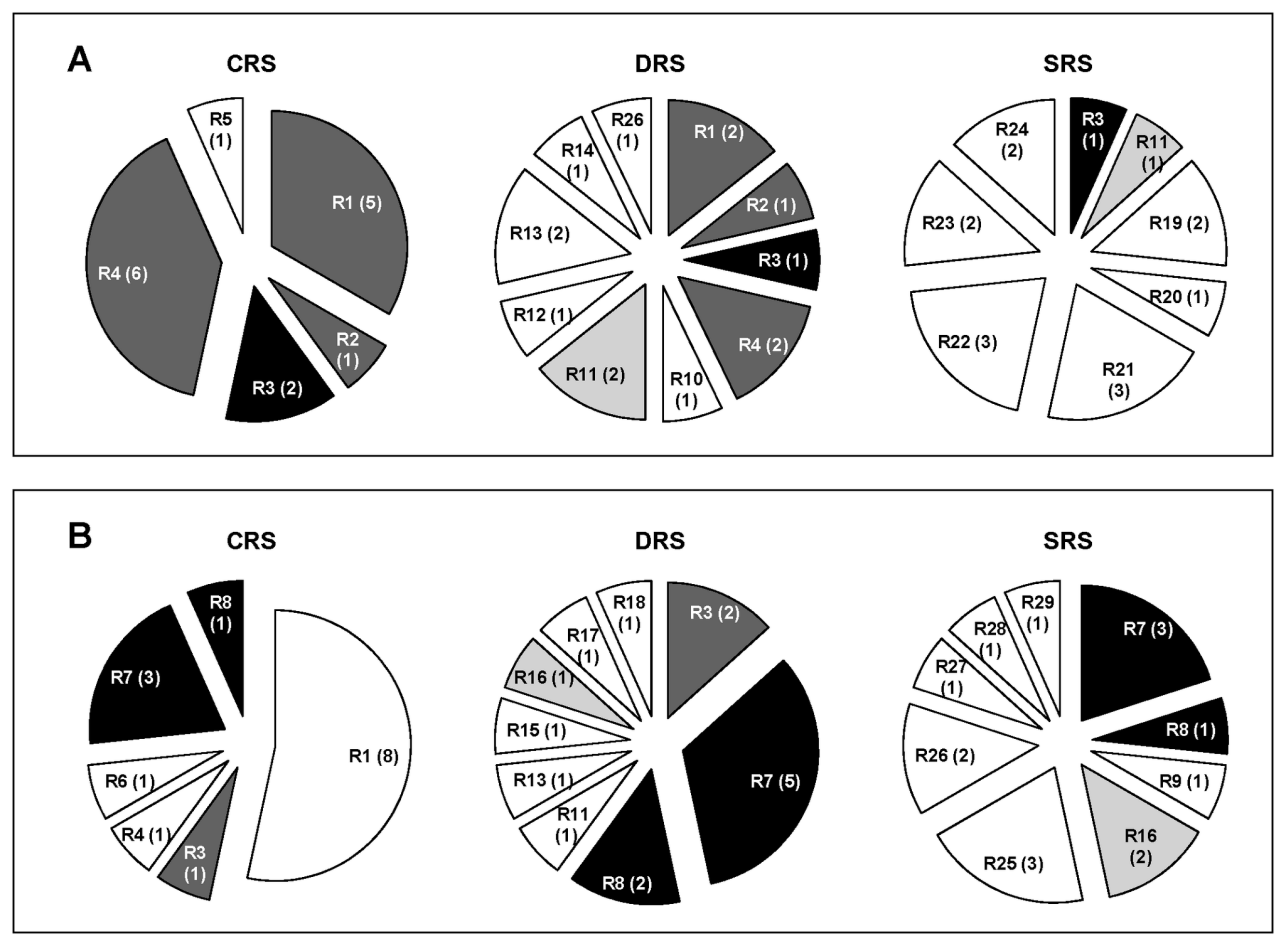
Distribution of ribotypes for HBFA (A) and LBFA (B) strains isolated from 3 types of alfalfa rhizospheric soil. Each "slice" corresponds to a particular restriction profile obtained by digestion of amplified *16S* *rRNA* gene (ribotype; R) with restriction endonuclease HaeIII. The number in parentheses is the number of strains that shared the ribotype for the particular treatment. Unique ribotypes are indicated by white. Shared ribotypes are indicated by light gray, dark gray, or black.

 In regard to the relationships between bacterial community structure, exposure to water-limiting conditions, and BFA of the strains, we observed clear differences among the ribotypic compositions of the cultivable bacterial communities in the 3 rhizospheric soils (CRS, DRS, SRS) (Figure 5). In general, both the HBFA and LBFA subpopulations from the rhizospheric soils subjected to stress conditions (DRS, SRS) had more restriction profiles (ribotypes) than those from nonstressed soil (CRS), suggesting the occurrence of diversification processes under stress conditions. Of the 15 HBFA strains in each treatment, we detected 5 ribotypes for CRS, 10 ribotypes for DRS, and 8 ribotypes for SRS (Figure 5A). Of the 15 LBFA strains in each treatment, we found 6 ribotypes for CRS, 9 ribotypes for DRS, and 9 ribotypes for SRS (Figure 5B). These findings appear to be inconsistent with those from a study of bacterial communities associated with the rhizosphere of canola (*Brassica napus*), in which a correlation was found between bacterial diversity and monthly rainfall [102]. However, comparisons between these studies are problematic because of differences in experimental design, soil type, and plant species.

 ARDRA revealed the presence of groups that are likely to compete with each other for establishment in the alfalfa rhizosphere because they are present under all 3 experimental conditions (R3 for HBFA, Figure 5A; R7 and R8 for LBFA, Figure 5B). We may speculate that under stressful conditions, a bacterial mixed biofilm formed by a more diverse community establishes itself in areas close to alfalfa roots as a consequence of the search for less hostile microenvironments. In contrast, under nonstressful conditions, the mixed biofilm community may be less diverse because the rhizosphere is colonized through establishment of more effective (i.e., competitive) bacterial species, reducing the probability that other species will settle in that microniche. An alternative explanation is that some bacterial species are able to survive under nonstressful conditions outside the rhizospheric microenvironment.

 Many previous studies have made comparisons of soil bacterial communities in relation to agricultural practices [57,103], soil types [104,105], soil history [106], and crop or plant species [107,108]. The present study is the first to describe the application of an abiotic stress type (water limitation) to a particular soil and the consequent structural changes of bacterial communities associated with the rhizosphere of a single plant species (*M. sativa*).

 Interestingly, the numbers of unique restriction profiles were larger for the stressful treatments (DRS, SRS; Figure 5). Within each treatment, most of ribotypes obtained for the HBFA subpopulation were different from those of the LBFA subpopulation. Three of 8 ribotypes (R1, R3, R4) were found in both subpopulations of the CRS group, whereas 3 of 15 ribotypes (R3, R11, R13) were found in both subpopulations of the DRS treatment. There were no shared ribotypes between the two subpopulations of the SRS treatment. These findings reinforce the concept that stressful conditions promote increased diversity of the members of the rhizospheric community.

**Figure 6:**
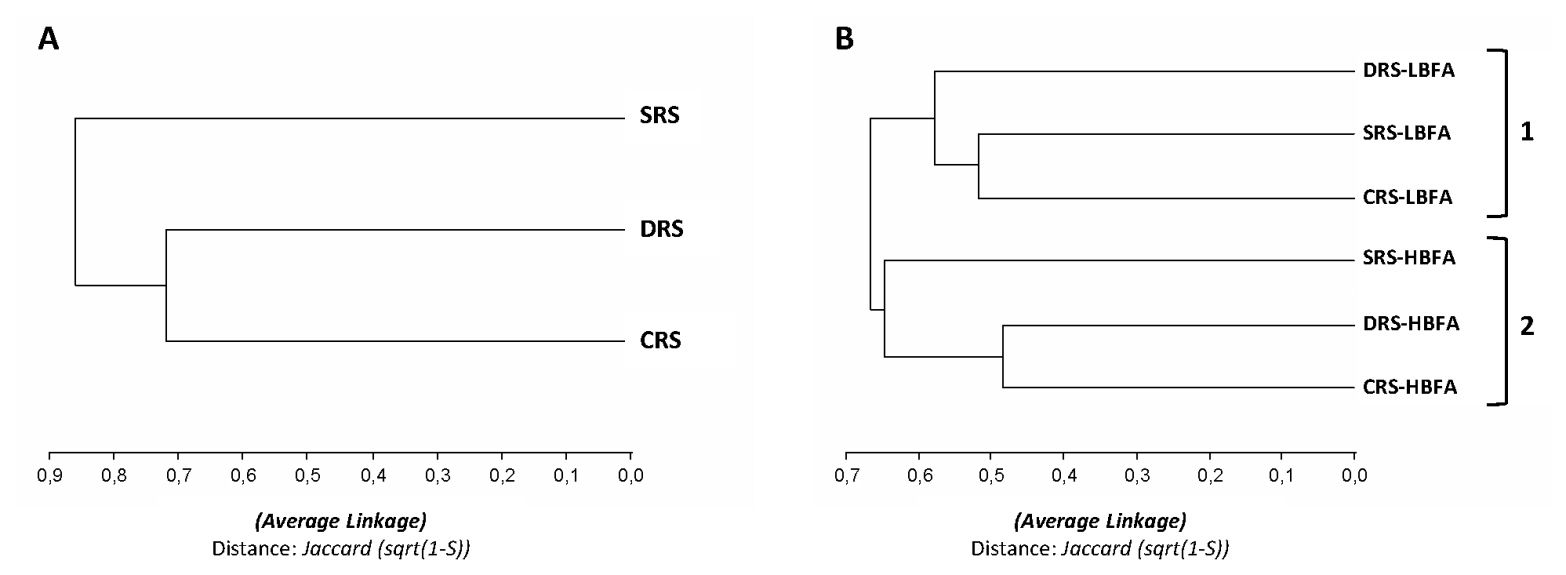
Dendrograms based on RFLP of *16S rRNA* gene analysis using the UPGMA algorithm. A: Dendrogram generated for the 3 types of alfalfa rhizospheric soil according to the ribotypes of the strains isolated. B: Dendrogram generated for the 3 types of soil in combination with the BFA (LBFA vs. HBFA) of the bacterial subpopulations, according to the ribotypes of the strains isolated.

 To further evaluate the differentiation among the 3 treatments and the subpopulations, we constructed a dendrogram with clustering according to the ribotypes found (Figure 6). When all ribotypes found for each condition were considered, the treatments clustered at a high distance (greater than 70%) (Figure 6A), indicating that the composition of bacterial rhizospheric communities varies depending on the exposure of soils to nonstressful conditions (CRS) or to water limitation via desiccation (DRS) or high salinity (SRS). Interestingly, the UPGMA dendrogram based on the ARDRA showed separation at a high distance of the LBFA and HBFA subpopulations into 2 clusters (groups 1 and 2, respectively; Figure 6B), reflecting the difference of the bacterial groups that constitute the 2 subpopulations. These findings suggest a link between physiological characteristics (BFA) and genetic characteristics.

### Identities and phylogenetic affiliations of bacterial strains from the 3 types of alfalfa rhizospheric soil

 Representative bacterial strains were identified by complete nucleotide sequencing of the *16S rRNA* gene. Strains were selected as belonging to majority or shared ribotypes according to their ARDRA profiles. The *16S rRNA* genes amplified with specific primers were of uniform size (~1400 bp). GenBank accession numbers and identities of the strains studied are shown in Table 1. The ARDRA ribotypes selected reflect the identity of the isolated strains with common Gram-positive and Gram-negative bacteria that are typically found in soils (Table 1) and interact with plants. These findings are consistent with those of previous studies [61,109,110].

 Interestingly, most of the strains from the HBFA subpopulations were identified as α-Proteobacteria or γ-Proteobacteria, whereas those from the LBFA subpopulations were identified as Actinobacteria. In contrast, some previous studies found that the dominant species in soil biofilms were Gram-negative [10,55,111]. However, these previous studies focused on the early formation of biofilms. We identified Proteobacteria as members of the HBFA subpopulations, which would most likely be the first to establish a biofilm. In regard to the rhizospheric microenvironment and in agreement with previous studies, we observed in the alfalfa rhizosphere the presence of bacterial groups (α- and γ-Proteobacteria, Actinobacteria) that are able to respond to root rhizodeposition, including rhizobia [69], *Pseudomonas* [38], and various Actinobacteria [112]. Our results are in contrast to those from studies of rhizospheric soils of nonlegume plants, in which β-Proteobacteria, Acidobacteria, and bacilli were the dominant groups [113]. These associations presumably depend on several factors, including plant type, that determine the composition of the microbial community that becomes established on the plant roots.

 The analysis of shared ribotypes demonstrated that certain members of both the HBFA and LBFA subpopulations are capable of colonizing the rhizospheric microenvironment of alfalfa regardless of stress condition. Ribotype 3 was present in the HBFA subpopulations for all 3 conditions and was associated primarily with α-Proteobacteria (strains C12 and S13, family Rhizobiaceae) and γ-Proteobacteria (strain M1, family Xanthomonadaceae). Ribotype 7 included members of the LBFA subpopulations belonging to the Actinobacteria, particularly genus *Arthrobacter*. These findings suggest that the presence of shared ribotypes with similar identities among the 3 treatments was ascribable to bacterial strains capable of establishing a mixed biofilm in the alfalfa rhizosphere, based on the ability to colonize the root environment (in the case of HBFA strains) or the opportunistic ability to become associated with mixed microconsortia (in the case of LBFA strains). Gram-negative bacteria may be primarily responsible for niche colonization and biofilm formation, whereas Actinobacteria simply take advantage of the biofilm structure to live in a protected microenvironment.

 In regard to the affiliations of unique majority ribotypes, we found identity of C3 (R1) with the genera *Agrobacterium* and *Rhizobium*, S2 (R19) with *Pseudomonas*, and S37 (R25) with *Shinella*. The identity of rhizospheric strains with members of the Rhizobiaceae, e.g., *Rhizobium* and *Shinella* [114], is interesting in view of the ability of these bacteria to establish symbiotic interactions with legumes. The nodulation ability of C3, C12, S13, and S37 was evaluated through inoculation of these strains on surface-sterilized seeds of *M. sativa* by the method described in our previous study [115]. Results of the nodulation tests were negative for all plants inoculated with the above strains, whereas positive results were obtained for plants inoculated with *E. meliloti* strain Rm 1021 (positive control) (data not shown). Regardless of these findings, it is remarkable that strains phylogenetically related to the rhizobia were present as part of mixed biofilms in the bacterial community associated with the alfalfa rhizosphere. These strains were found in the CRS and SRS groups, suggesting a tolerance to water-limiting conditions resulting from salinity but not from desiccation.

 We examined the identity of strains with the R4 ribotype (C7, M10) that is a majority ribotype shared by the HBFA subpopulations of CRS and DRS. These strains were identified as members of the genus *Microbacterium*, indicating that some Actinobacteria have good BFA.

 Comparison of our findings for strain identity (Table 1) and ribotype composition (Figure 5) suggests that under nonstressful conditions (CRS) the bacterial community is less diverse and consists primarily of members of the Rhizobiaceae (R1) and Actinobacteria (R4, R7). Under water-limiting conditions (DRS, SRS), the communities are more diverse and consist primarily of unique ribotypes. Although shared members with similar phylogenetic affiliations (R3, R7, R8) are present, they are not the majority ribotypes in these communities.

 The presence of members of the Actinobacteria (particularly Actinomycetes) in biofilm microconsortia established in the alfalfa rhizosphere is interesting for several reasons: (i) the formation of mixed biofilms including Gram-positive (Actinobacteria) and Gram-negative (Proteobacteria) bacteria provides an interesting research model of biofilm development by bacteria of different phyla; (ii) the potential role of Actinomycetes as plant growth-promoting rhizobacteria (PGPR) [9,116,117] is poorly known in comparison to other PGPR, such as *Pseudomonas* and rhizobia; (iii) our knowledge of community structure and diversity of Actinobacteria in alfalfa rhizospheric soils is limited; (iv) Actinobacteria may play important ecological roles in the rhizosphere microenvironment.

 Because of the small number of strains sequenced, our results and interpretations as presented in this article represent only a restricted and partial view of a very complex and dynamic microenvironment. However, this study is the first to assess alfalfa rhizospheric communities as mixed biofilms in the context of water-limiting conditions, and our findings provide an important basis for more extensive studies in the future.

### BFA-related traits of bacterial strains from 3 types of alfalfa rhizospheric soils

 Biofilm formation is a multistep process that requires the integration of various bacterial physiological processes, including quorum sensing [118,119], motility [120,121], autoaggregation [34], and EPS production [73,122].

 We evaluated the BFA-related traits of the selected cultivable bacterial strains in relation to ribotype (Table 1). In general, the ability of the strains to produce BFA-related phenotypes was variable. The autoaggregative phenotype of the strains ranged from barely to highly aggregative types, with no apparent relationship to strain origin or phylogenetic affiliation. There was also no obvious correlation between ribotype and EPS production. However, it should be noted that the nonspecific calcofluor fluorescence staining test used detects only the presence of β linkages among sugars, and the "negative" strains may have had EPS-producing ability that was not detected by calcofluor staining.

 Our evaluation of QS signal production showed that all of the tested strains were incapable of synthesizing short-chain AHLs (data not shown). Nine of the 13 isolates were able to produce long-chain AHLs according to our methodology, and no direct relationship was found between BFA and AHL production. Three of the 4 AHL-negative strains belonged to the LBFA groups and the other (M1) belonged to an HBFA group and was positive for the remaining BFA-related traits. These findings suggest that "cross-talk" information exchange may occur in the rhizospheric microenvironment, reflecting the complexity of interactions at the biofilm microconsortium level. As suggested previously [123], QS signals synthesized by certain members of taxonomically and functionally complex multispecies biofilms may coordinate the behavior of the entire community (microconsortium), with an overall beneficial effect. Our evaluation of QS signal production was limited to the detection of AHL-like molecules. This type of signal, which is typically produced by Gram-negative bacteria, was surprisingly detected in strains identified as Actinobacteria. This finding is of interest because QS mechanisms found to date in Gram-positive bacteria involve the production of small peptides. We did not test for the possible production of the autoinducer 2 (AI-2) signal, which has been reported to occur in both Gram-negative and Gram-positive bacteria [124].

 Our motility (swimming) assays indicated the presence of 3 groups: (i) non-motile strains, which were associated with LBFA groups identified as Actinobacteria, (ii) low-motility strains, regardless of phylogenetic affiliation or BFA, and (iii) high-motility strains, which were associated with HBFA strains identified as Proteobacteria (Table 1).

 To further integrate and provide explanations for the relationships among BFA-related traits, ribotypes, phylogenetic affiliations, and experimental treatments, we performed a multivariate PCA. This analysis provides a graph that facilitates visualization and interpretation of the data set and the variables. For the PCA, the observations (cases) were the ribotypes (R) and phylogenetic affiliations (Actinobacteria, Proteobacteria) associated with different treatments (CRS, DRS, SRS) and subpopulations (HBFA, LBFA); the variables were the BFA, autoaggregation, motility, AHL production, and EPS production of each strain belonging to a given observation.

**Figure 7:**
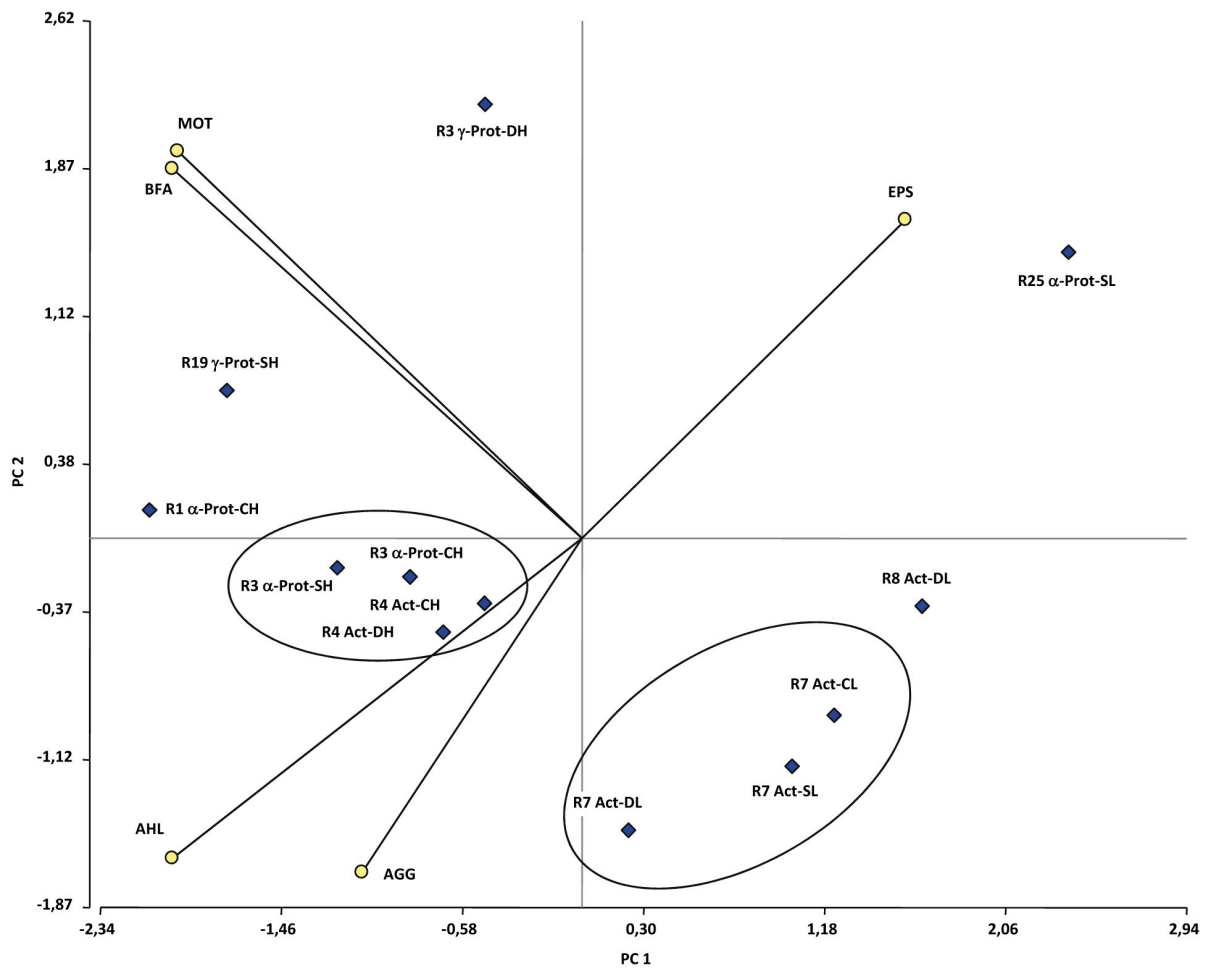
Relationships among BFA-related traits, ribotypes, phylogenetic affiliations, and experimental treatments. The graph was obtained from PCA using the InfoStat software program, version 2.0. Diamonds indicate combinations of ribotypes and affiliations with treatments. R: ribotype. Act: Actinobacteria. Prot: Proteobacteria. C: CRS. D: DRS. S: SRS. H: HBFA. L: LBFA. Circles indicate biological variables: BFA, biofilm-forming ability; MOT, motility; AGG, autoaggregation; AHL, production of QS signal; EPS, EPS production. The angles formed between the straight lines indicate the degree of correlation between variables (see text). PC1: Principal Component 1. PC2: Principal Component 2.

 The PCA applied to all data in 3 dimensions (PC1, PC2, PC3) explained ~86% of the total variability in the study. The graph generated from PC1 and PC2 (which explained ~70% of the variability) (Figure 7) indicates that no association could be made in relation to treatment. Thus, the selection of traits related to BFA-associated phenotypes was not affected by water-limiting conditions, which was obvious because HBFA and LBFA subpopulations were identified for all 3 treatments. There was a clear separation of observations into 2 groups as a function of phylogenetic affiliation and HBFA vs. LBFA status: (i) Actinobacteria with LBFA status (ribotypes R7 and R8, found for all 3 treatments), and (ii) Actinobacteria with HBFA status (ribotype R4, found for CRS and DRS) and most of the Proteobacteria. Group (i) was in contrast to the phenotypes with BFA-related traits and had some proximity to the autoaggregative phenotype. Such autoaggregative capability may allow these strains to join an existing biofilm and take advantage of the traits of the other members. Group (ii) includes a subgroup of strains belonging to the α-Proteobacteria (R3) and Actinobacteria (R4) that display similar behaviors in regard to BFA. The positioning of unique ribotypes such as R1 and R19 next to this subgroup is interesting. These associations of strains with differing phylogenetic affiliations reflect the complexity of interactions possible in multispecies biofilms established as a community in the alfalfa rhizosphere. The positioning of γ-Proteobacteria ribotype R3 (identified as strain M1), which was not closely associated with members of group (ii), is interesting because this strain does not belong to the α-Proteobacteria and has strong BFA-related traits. Another unique ribotype, R25 (strain S37), is characterized by being the most distant in relation to both other groups and BFA-related traits (Table 1; Figure 7).

 In regard to the associations among the variables, we found a strong positive correlation (acute angles in Figure 7) between the variables of motility and biofilm formation. This finding is consistent with previous evidence that the initial step of colonization and the subsequent developmental steps of biofilm formation depend on differing bacterial motility mechanisms [125]. Biofilm formation showed weaker correlations (more open angles in Figure 7) with AHL production and autoaggregation. One possible interpretation of this finding is that bacteria, prior to physical contact for cell aggregation, must communicate, move, and then interact among themselves and with nearby surfaces.

 Surprisingly, no associations (i.e., right or obtuse angles in Figure 7) were found among EPS production, biofilm formation, AHL production, and autoaggregation, suggesting that these bacterial mechanisms may be regulated and used differently as determined by environmental or plant signals. It is also possible that other EPSs and/or QS signals not evaluated in this study play important roles in the complex process of a multispecies community development.

 Improved knowledge of traits in bacterial strains that confer increased survival and plant protection against environmental stressors will certainly be useful for management of agricultural practices. Morphological, physiological, and molecular approaches for elucidation of bacterial mechanisms that enhance tolerance of stress conditions will help us obtain this knowledge.

## Conclusions

 The findings presented here demonstrate that the rhizospheres of *Medicago sativa* (alfalfa) plants exposed to differing water-limiting conditions harbor distinct bacterial communities (microconsortia) with differing abilities to develop biofilms and thus to establish themselves in this microenvironment. Judging by observed changes in colony sizes (counts), WBFA, and community structures, the ecological functions of rhizospheric biofilm microconsortia vary depending on exposure to stressful conditions, presumably to enhance bacterial community survival, plant growth, and protection from the stress conditions. We found that rhizospheric soils exposed to desiccation conditions (DRS) contained bacterial communities with higher WBFA in comparison to those exposed to saline stress (SRS) or no water limitation (CRS). Our results indicate that water-limitation stress led to selection of bacterial strains in the alfalfa rhizospheric niche that employed the protected biofilm microenvironment as a strategy to survive in the dry soil.

 Ribotyping analysis based on ARDRA showed that bacterial communities present in the 3 types of soils (CRS, DRS, SRS) were strongly differentiated. Such heterogeneity was present between the HBFA vs. LBFA subpopulations in a given type of soil, accentuating the separation of the communities.

 The phylogenetic affiliation analysis of the selected strains showed that Actinobacteria and Proteobacteria are the predominant members of alfalfa rhizospheric microconsortia. Certain bacterial ribotypes were present in the alfalfa rhizosphere regardless of exposure to stressful conditions. On the other hand, exposure to the 2 water-limiting conditions (DRS, SRS) resulted in the appearance or disappearance of other ribotypes, suggesting that such stress is a key factor that modulates the physiology of the host plant, bacterial species, and rhizospheric community. The composition of alfalfa rhizospheric microconsortia appears to be strongly affected by interaction phenomena such as synergism, antagonism, cooperation, and opportunism, as evidenced by the presence in this edaphic niche of bacterial strains with differing BFA.

 In summary, the present findings improve our understanding of the structure and physiology of bacterial communities established as biofilm microconsortia in the rhizosphere of alfalfa, an important forage legume worldwide, exposed to differing water-limiting environments. These data provide a basis for further studies of the members of these communities at many levels (ranging from molecular to ecological) that will lead to effective new tools for improved management of agricultural practices and increased understanding of these crucial microorganisms.
